# Prospective evaluation of an AI-enabled elastic scattering spectroscopy device for triage of patient-identified skin lesions in dermatology clinics

**DOI:** 10.1016/j.jdin.2025.07.007

**Published:** 2025-08-22

**Authors:** Erik Jaklitsch, Selina Chang, Sabrina Bruno, Nicholas D’Angelo, Joe K. Tung, Laura K. Ferris

**Affiliations:** a University of Pittsburgh School of Medicine, Pittsburgh, Pennsylvania; b Department of Dermatology, University of Pittsburgh, Pittsburgh, Pennsylvania; c Department of Dermatology, University of North Carolina, Chapel Hill, North Carolina

**Keywords:** artificial intelligence, basal cell carcinoma, diagnostic tools, elastic scattering spectroscopy, machine learning, melanoma, skin cancer, squamous cell carcinoma, technology

*To the Editor:* Early detection of skin cancer holds promise for reducing mortality and surgical morbidity.^1^ However, wait time for suspicious lesions can be long, and although many patient-identified lesions are benign, initial skin cancer presentations are often first noticed by patients.^2^^,^^3^ A highly sensitive Elastic Scattering Spectroscopy (ESS) device integrated with artificial intelligence (AI) utilizing spectral scans was cleared for adjunct usage in primary care and is the only FDA-cleared tool for evaluating all 3 common types of skin cancer (melanoma, basal cell carcinoma [BCC], and squamous cell carcinoma [SCC]).

This investigator-initiated, matched comparison study aims to evaluate device performance on patient-identified lesions in a dermatology setting to assess its utility for screening lesions appropriate for referral across clinical contexts. We prospectively evaluated 150 patients presenting with self-identified lesions of concern to 3 outpatient dermatology offices. Diagnostic accuracy of device readout (classifying lesions as either “Monitor,” alone, or “Investigate Further,” alongside a score of 1 to 10, indicating spectral similarity to malignant tissue) was compared to blinded dermatologist management decisions and histopathology.

The study included 150 lesions in 72 males and 78 females, with a mean age of 59.6 years. Malignant lesions were identified in 14.7% of cases (22/150), with melanoma, BCC, and SCC accounting for 3, 9, and 10, respectively (Table I). The ESS device identified 138 lesions as “Investigate Further” and 12 as “Monitor.” Sensitivity for detecting malignant lesions was 100%, negative predictive value (NPV) was 100%, specificity was 9.4%, and positive predictive value (PPV) was 15.9%. For lesions with a spectral score of 7 to 10, sensitivity was 86.4%, NPV 96.6%, with specificity increasing to 67.2% and PPV to 31.1%. Compared to dermatologists' decisions, sensitivity was 95.5%, NPV 75%, specificity 10.7%, and PPV 45.7% (Table II). Overall AUC of the device compared to malignant lesions was 0.79 (Supplementary Fig 1, available via Mendeley at https://data.mendeley.com/datasets/vpxw84996p/1), identical to a validation study in primary care.^4^ Frequency counts of benign and malignant lesions compared to corresponding spectral score are also visualized (Supplementary Fig 2, available via Mendeley at https://data.mendeley.com/datasets/vpxw84996p/1).

**Table I:** Patient demographics, research for concern, lesion location, classification, and dermatologist management decision

Patient demographics	*n* (%)
Sex (male)	72 (48.0)
Sex (female)	78 (52.0)
Age (mean ± SD; range)	59.6 ± 17.5; 22-92
Reason for concern	*n* (%)
Worry for skin cancer	120 (80.0)
Causes discomfort	15 (10.0)
Aesthetic concern	8 (5.3)
Other	7 (4.7)
Lesion location	*n* (%)
Trunk	50 (33.3)
Head	42 (28.0)
Upper extremity	19 (12.7)
Lower extremity	23 (15.3)
Other	16 (10.7)
Lesion classification	*n* (%)
Melanoma	3 (2.0)
Basal cell carcinoma	9 (6.0)
Squamous cell carcinoma	10 (6.7)
Actinic keratosis	15 (10.0)
Seborrheic keratosis	57 (38.0)
Dermatofibroma	5 (3.3)
Nevus	34 (22.6)
Other	17 (11.3)
Dermatologist management	*n* (%)
Biopsy	66 (44.0)
Treat	31 (20.7)
Monitor	13 (8.7)
Reassurance	40 (26.7)

**Table II:** Sensitivity, specificity, NPV, and PPV as compared to histopathology and dermatologist decision to biopsy

	Compared to pathology (benign vs malignant, 0 score negative)	Compared to pathology (1-2 score negative)	Compared to pathology (1-6 score negative)	Compared to derm decision to biopsy
Overall sensitivity	100% (22/22)	100% (22/22)	86.4% (19/22)	95.5% (63/66)
Melanoma sensitivity	100% (3/3)	100% (3/3)	66.7% (2/3)	
BCC sensitivity	100% (9/9)	100% (9/9)	88.9% (8/9)	
SCC sensitivity	100% (10/10)	100% (10/10)	90% (9/10)	
Overall specificity	9.4% (12/128)	21.9% (28/128)	67.2% (86/128)	10.7% (9/84)
BMN specificity	8.8% (3/34)	26.5% (9/34)	79.4% (27/34)	
SK specificity	14% (8/57)	28.1% (16/57)	68.4% (39/57)	
Benign other specificity (including AK)	2.7% (1/37)	8.1% (3/37)	54.1% (20/37)	
AK specificity	0% (0/15)	0% (0/15)	26.7% (4/15)	
Benign other specificity (excluding AK)	4.5% (1/22)	13.6% (3/22)	72.7% (16/22)	
Overall NPV	100% (12/12)	100% (28/28)	96.6% (86/89)	75.0% (9/12)
Overall PPV	15.9% (22/138)	18% (22/122)	31.1% (19/61)	45.7% (63/138)

These results demonstrate that the ESS device maintains robust sensitivity and NPV for detecting malignant lesions, even in a dermatology setting with patient-identified lesions, and support its role as an adjunctive rule-out tool in skin cancer triage. Despite a relatively low specificity (9.4%) and PPV (15.9%), most false positive results (59.5%) were actively managed by dermatologists through biopsy or treatment. Furthermore, given dermatologist assessment was used as the gold standard, all unbiopsied lesions had a biased specificity of 100%. Spectral score was a reliable positive predictor of malignancy, with these results (using a threshold of spectral score 7 to 10 for tissue of the highest similarity to malignant tissue) nearly identical to a prior study.^5^

Though limited by sample size and a single dermatologist reference, this study is unique in including only patient-selected lesions, with outcomes compared to dermatologists’ management decisions. This study suggests that the ESS device, as a complementary tool, may enhance triage and decision-making for patient-identified lesions to be employed by non-specialists in any clinical setting. Still, physicians using the device should utilize appropriate clinical context to minimize unnecessary interventions.

## Conflicts of interest

None disclosed.
